# Chemical- and green-precursor-derived carbon dots for photocatalytic degradation of dyes

**DOI:** 10.1016/j.isci.2024.108920

**Published:** 2024-01-17

**Authors:** Inderbir Kaur, Vandana Batra, Naveen K.R. Bogireddy, Jasmina Baveja, Y. Kumar, V. Agarwal

**Affiliations:** 1 Department of Electronic Science, Bhaskaracharya College of Applied Sciences, University of Delhi, Delhi, India; 2 Department of Physics, Bhaskaracharya College of Applied Sciences, University of Delhi, Delhi, India; 3 ICF-UNAM, Av. Univ. 1001, Col. Chamilpa, Cuernavaca, Morelos 62209, Mexico; 4 Invited Researcher at Center for Research in Engineering and Applied Sciences (CIICAp-IICBA), Autonomous State University of Morelos (UAEM), Av. Univ. 1001, Col. Chamilpa, Cuernavaca, Morelos 62209, Mexico; 5 Departamento de Fisico Matematica, UANL, Monterrey, Mexico; 6 Center for Research in Engineering and Applied Sciences (CIICAp-IICBA), Autonomous State University of Morelos (UAEM), Av. Univ. 1001, Col. Chamilpa, Cuernavaca, Morelos 62209, Mexico

**Keywords:** Chemistry, Chemical engineering, Materials science, Materials chemistry

## Abstract

Rapid industrialization and untreated industrial effluents loaded with toxic and carcinogenic contaminants, especially dyes that discharge into environmental waters, have led to a rise in water pollution, with a substantial adverse impact on marine life and humankind. Photocatalytic techniques are one of the most successful methods that help in degradation and/or removal of such contaminants. In recent years, semiconductor quantum dots are being substituted by carbon dots (CDs) as photocatalysts, due to the ease of formation, cost-effectiveness, possible sustainability and scalability, much lower toxicity, and above all its high capacity to harvest sunlight (UV, visible, and near infrared) through electron transfer that enhances the lifetime of the photogenerated charge carriers. A better understanding between the properties of the CDs and their role in photocatalytic degradation of dyes and contaminants is required for the formation of controllable structures and adjustable outcomes. The focus of this review is on CDs and its composites as photocatalysts obtained from different sustainable green as well as chemical precursors. Apart from the synthesis, characterization, and properties of the CDs, the study also highlights the effect of different parameters on the photocatalytic properties of CDs and their composites for catalytic dye degradation mechanisms in detail. Besides the present research development in the field, potential challenges and future perspectives are also presented.

## Graphical abstract

**Figure:**
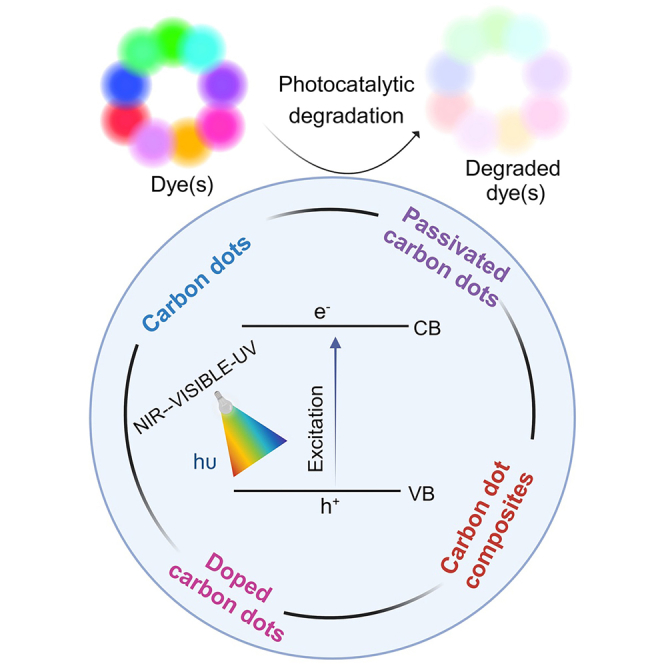

## Introduction

Industrial growth has resulted in an era of excessive utilization and handling of hazardous chemicals and organic pollutants. Toxic and carcinogenic wastes, dyes, chemicals, and pollutants are discharged into the environment without proper treatment. Dumping these untreated industrial effluents leads to air, soil, and water pollution, causing severe health concerns for plant, aquatic, and human life. In particular, dye effluents from industries like textile, leather, tannery paint, food processing, and cosmetics block sunlight penetration, reduce oxygen dissolution, and interrupt photosynthesis in aquatic plants, leading to a significant threat to marine life. The above-mentioned hazardous contaminants must be eliminated or degraded into less harmful compounds before their discharge into environmental water. As the prevalent traditional dye reduction/removal technologies are costly and add secondary environmental pollution, developing new practical methods/solutions for removing these toxic dyes is crucial.^1^^,^^2^^,^^3^^,^^4^^,^^5^^,^^6^^,^^7^^,^^8^ Various techniques like ion exchange, adsorption, photo-Fenton process, advanced oxidation processes (AOPs), photocatalytic degradation, membrane filtration, and electrochemical treatments are conventionally used in this direction, with photocatalytic degradation being the most preferred approach due to its excellent efficiency and moderate reaction conditions.^1^^,^^7^^,^^9^

Although various semiconductor photocatalysts with different morphologies have been explored, most of them are found to be toxic, time-consuming, economically less viable, involve complex synthesis steps, and/or show comparatively less activity in the visible range^3^^,^^7^^,^.^10^^,^^11^^,^^12^^,^^13^^,^^14^^,^^15^^,^^16^^,^^17^^,^^18^ On the other hand, recently developed economically viable and scalable technology^19^ of carbon quantum dots (CQDs) has attracted attention due to their biocompatibility, excellent photo-responsiveness (high capacity to harvest UV, visible, and near infrared light), less toxicity, more stability, easy tunability, and good aqueous solubility,^20^^,^^21^ with reusability in different supported systems (composites) and hence applicability in different areas.^22^^,^^23^ They also have many unique optical characteristics including excellent photostability and upconversion capability.^24^ Their ability to absorb light throughout the spectrum enables them to degrade numerous contaminants on irradiation with visible light. Based on the synthesis techniques of CDs, various functional groups containing oxygen are present on the amorphous shell covering the sp^2^/sp^3^ conjugated core.^25^ Surface carboxylic acid moieties improve their solubility in water,^26^^,^^27^ which is an essential requirement for its application as a photocatalyst. Apart from the enhanced contact area between CDs and contaminants owing to their small dimensions and the presence of various functional groups on the surface of CDs, the surface states on CDs may act as electron scavengers, leading to the inhibition of electron-hole pair recombination causing the charges to transfer efficiently, which is the ideal requirement for photocatalysis.^14^^,^^28^

CDs doped with metals/non-metals (N, B, etc.), and their composites with oxides like TiO_2_, tungsten oxide (WO_2_), ZnO, zinc sulfide (ZnS), strontium peroxide (SrO_2_), and cadmium sulfide (CdS) are usually employed for enhanced stability, photocatalytic activity, extending the light harvesting ability as well as reusability.^29^^,^^30^^,^^31^ Due to the enhanced surface area and charge carrier mobility, the nanocomposites have been reported to possess improved dye degradation.^32^

In addition, although photocatalytic degradation of dyes has been achieved using chemically synthesized CDs, the degradation using green-precursor-derived CDs is being preferred due to their biocompatibility, environment-friendly/non-toxic nature, and cost-effectiveness.^33^^,^^34^^,^^35^^,^^36^^,^^37^^,^^38^^,^^39^ This review deals with the recent development of the application of chemical as well as green-precursor-derived CDs in the photocatalytic degradation of various toxic dyes and will serve as a reference for future developments in the field of dye degradation. The current study consists of eight sections in all. The first section (introduction) reviews the necessity for the degradation of dyes and the importance of CDs, second section introduces various approaches for synthesizing CDs, third section gives an overview of their structural and optical characterizations, fourth section gives brief introduction to different dyes and their harmful effects and compares and summarizes chemical and green-precursor-based CDs used in photocatalytic degradation of dyes, fifth section summarizes the various parameters that affect the photocatalytic mechanism, sixth section gives a brief overview of theoretical tool to guide catalyst design, seventh section describes dye degradation mechanisms, and finally eighth section sums up the conclusion followed by future perspectives discussing the milestones achieved and the challenges yet to be tackled for the application of chemical and green CDs toward the degradation of harmful dyes.

## Synthesis of carbon dots

**Figure 1:**
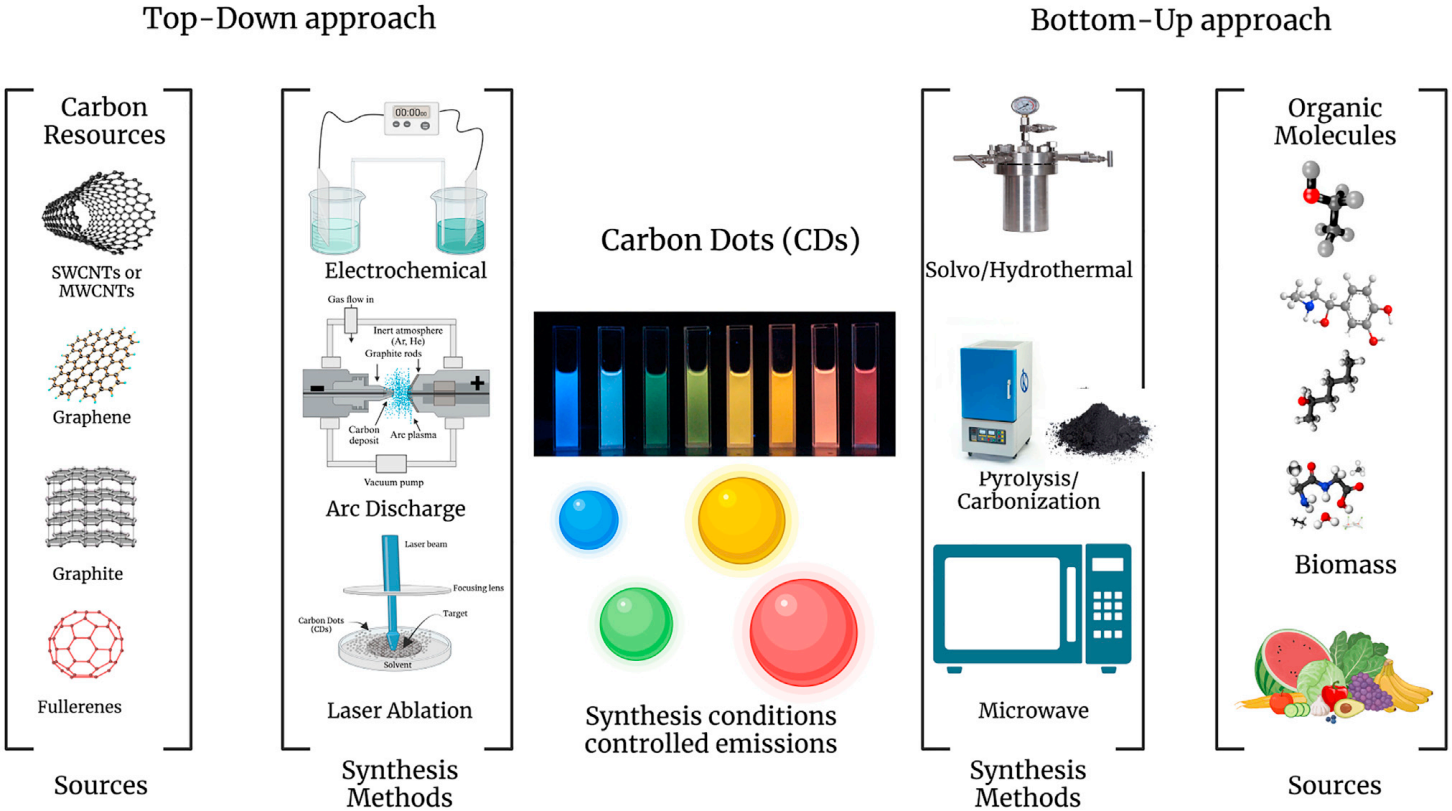
An overview of widely used top-down and bottom-up approaches for the synthesis of CDs, using green and chemical precursors

Numerous techniques for synthesizing CDs have been developed, where top-down and bottom-up are the two primary categories for the same^40^ (Figure 1). The top-down method involving the physical or chemical breakdown of large molecules into CDs of varying sizes includes approaches such as laser ablation,^41^ arc discharge, chemical oxidation, electrochemical oxidation, plasma treatment, etc. In contrast, the bottom-up method focuses on small-molecule chemical polymerization and carbonization, through techniques such as hydrothermal/solvothermal, thermal decomposition, microwave, pyrolysis, etc.^25^^,^^42^

The most prevalent methods to synthesize CDs i.e., **hydrothermal**, **solvothermal**, and **carbonization,** are relatively inexpensive, nonhazardous, and environment-friendly. In the hydrothermal technique (water used as solvent), with an application of high vapor pressure and temperature, precursors get carbonized within the aqueous solution to form water-soluble CDs. These CDs have been produced using different raw materials, such as proteins, citric acid, natural green sources, biowaste such as Pomelo (*Citrus maxima* or *Citrus grandis*) juice, *Salvia Hispanica L.* (Chia), empty fruit bunches (Palm oil), etc.^5^^,^^14^^,^^36^^,^^43^

Typically, hydrothermal synthesis of CDs is performed by sealing a solution of the organic or inorganic precursors in a reactor and heating it between 150°C and 200°C. Different reagents can be added to the synthesis reaction to modify the surface functional groups of CDs. For instance, Komalavalli et al.^44^ showed the formation of hydroxyl-functionalized fluorescent CDs using hydrothermal treatment of *Hibiscus Sabdariffa* leaves at 160°C for 8 h. Mehta et al.^45^ reported a green hydrothermal route for the plant-based synthesis of *Saccharum-officinarum*-juice-derived CDs. These CDs served as excellent fluorescent probes for *Escherichia coli* and yeast cellular imaging. Hydrothermal carbonization transformed organic matter into CDs and introduced surface functionalization, making the CDs photoluminescent and water soluble.^46^ Orange-peel- and turmeric-derived CDs were hydrothermally treated at 180°C and 200°C for 12 h and at 24 h, to obtain CDs with high quantum yields (QYs) as 36% and 20%, respectively.^18^^,^^37^

In the case of the **solvothermal** method, various solvents, including ethanol and N, N-dimethylformamide (DMF), are utilized.^47^ For example, Jiang et al.^48^ synthesized three different types of CDs with red, green, and blue photoluminescence (PL), using isomers of phenylenediamines, as precursors, and ethanol as solvent under similar conditions of temperature and pressure. Similarly, Zhi et al.^49^ obtained polymeric CDs with multiple colors by combining citric acid and urea in formamide and have shown QYs ranging from 9.8% to 60%, depending on the color.

The widely scalable and environment-friendly synthesis of CDs was made possible using natural precursors. For example, pear juice was hydrothermally treated for 36 h at 180°C, which was separated using a 0.2 μm membrane.^20^ Using rice bran, fluorescent CDs are synthesized at 200°C for 4 h with no surface passivation.^20^ Fish scales, a nitrogen-rich waste product that includes chitin and collagen, were utilized for generating CDs, without using any other reagent.^50^ Various proportions of sodium hydroxide (NaOH), fluorine, and nitrogen concentrations have also been used to generate green-, yellow-, and orange-emitting CDs, taking gourd flesh as a carbon and fluorine precursor and urea for nitrogen source.^1^

In addition, there have been numerous reports on the synthesis of CDs/semiconductor nanocomposites used for the enhancement of photocatalytic behavior.^51^ Using a low-temperature hydrothermal method, CDs (blue emitters) were synthesized from coffee powder, which were further immobilized on ZnO nanoparticles to form ZnO/CD nanocomposite.^52^ Further, Yu et al.^51^ fabricated CQDs/TiO_2_ nanosheet (TNS) composites, which displayed enhanced photocatalytic properties as compared with pure TNS. Similarly, dark brown colored nanocomposites with TiO_2_ nanoparticles^6^ and ZnO nanoparticles^7^ were synthesized using peach fruit juice as a carbon source. Using corn powder, the graphene quantum dots (GQDs) and their composite with TiO_2_ were developed by researchers using hydrothermal and *in situ* sonochemistry methods, respectively.^53^ CDs/ZnO^54^ at room temperature and ZnO foam/CQDs^55^ at 80°C were also synthesized for similar application in dye degradation.

While preparing magnetic CD composites, i.e., synthesizing CDs on the Fe_3_O_4_ surface at 140°C, the saturation magnetization was found to decrease with an increase in reaction time.^56^ Hydrothermal treatment of graphene oxide, treated at 200°C for 4 h generated multi-layer GQDs.^57^ In addition, the **microwave-assisted** technique has also been established as one of the straightforward, inexpensive, quick, clean, versatile, and high-yield methods for producing CDs through the direct carbonization of organic compounds.^58^^,^^59^^,^^60^^,^^61^^,^^62^ In this technique, molecular-level interaction with applied alternating voltage in solvent results in heat at the molecular level.^63^^,^^64^^,^^65^ As some specific examples of microwave-assisted procedures, Zhou et al.^66^ used citric acid and 1,2-phenylenediamine as carbon source and nitrogen dopant, respectively. Further, CDs/ZnO, having *Gum Ghatti*, an *Anogeissus latifolia* tree discharge, and zinc acetate as precursors, are synthesized using microwave pyrolysis.^3^ Using green precursors like *aloe vera* with microwave-assisted reflux method^21^ and *Bougainvillea* leaves extract, using a household microwave,^67^ blue- and red- emitting CDs are synthesized, respectively. Due to the quick reaction during microwave heating, the concentration of both B and P heteroatoms has been found to be greater in microwave-aided CDs (QY 52%), as compared with hydrothermal synthesis (QY 43%), which in turn explains its relatively higher QY, demonstrating the efficacy of microwave synthesis.^68^

In comparison to the methods discussed earlier, **thermal decomposition** is another simple, fast, and cost-effective technique to generate CDs where a material (organic molecular/polymeric precursor; single/multi-component) is decomposed chemically upon heat treatment, through endothermic reactions^69^^,^^70^ with several intermediates. Using this technique, citric acid is dehydrated and reduced in the temperature range of 180°C–200°C to generate CDs.^71^ However, **pyrolysis** or **carbonization techniques** are frequently used for synthesizing CDs under high temperature conditions, requiring strong acids/alkalis to convert precursors into nanoparticles.^72^^,^^73^^,^^74^

## Structural and optical characterization of carbon dots

### Electron microscopy, X-ray photoelectron spectroscopy, and infrared spectroscopy

**Figure 2:**
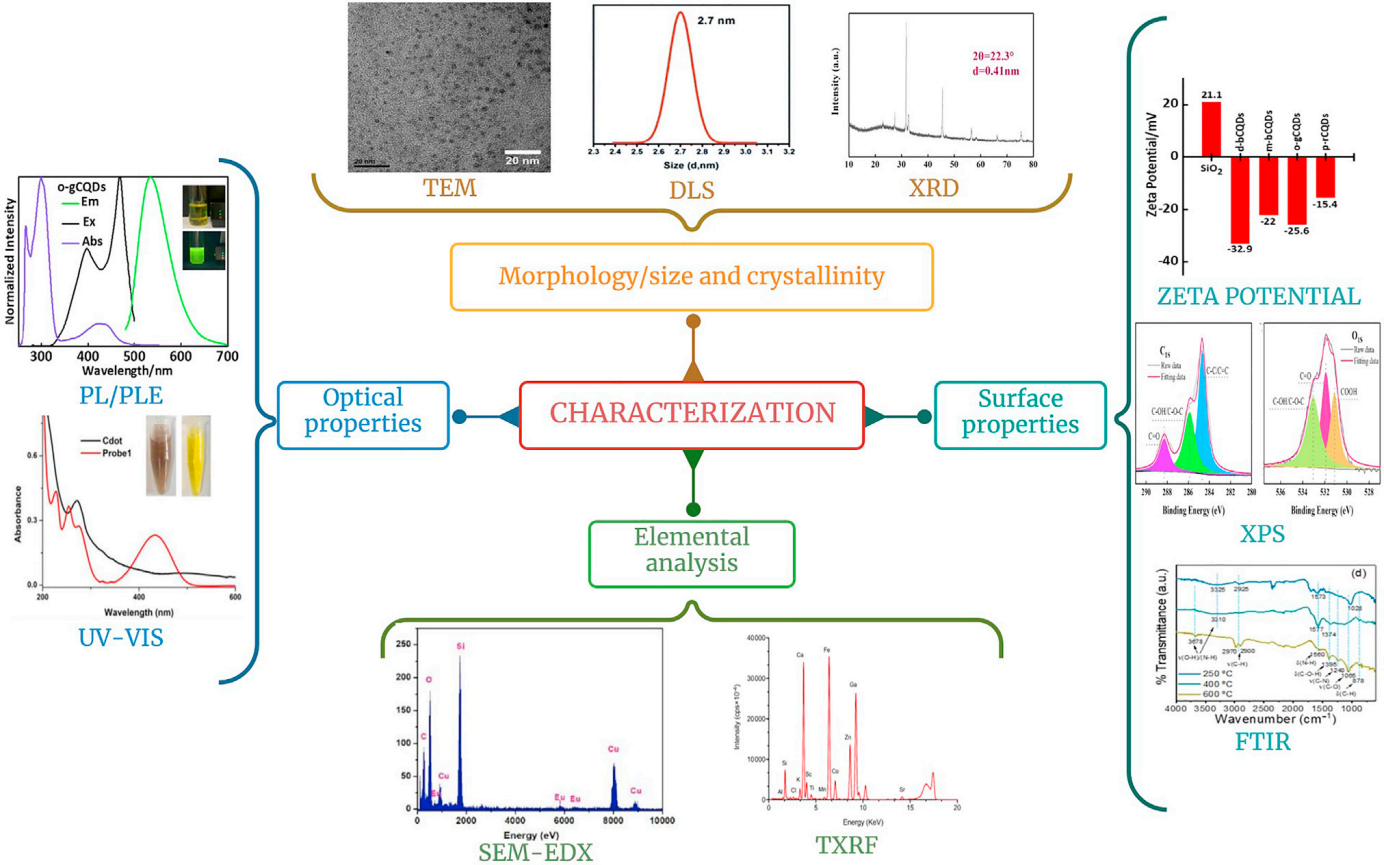
Various techniques used to determine structural and optical characteristics of carbon dots (CDs) TEM,^80^ FTIR,^80^ DLS,^81^ XRD,^82^ Zeta potential,^83^ XPS,^83^ PL/PLE,^83^ UV-Vis,^84^ SEM-EDX,^85^ and TXRF.^86^

The structure of CDs and its composites involves examining crystallinity, size, morphology, and presence of functional groups using a range of characterization techniques^72^^,^^75^ (Figure 2). One of the extensively used techniques is transmission electron microscopy (TEM), which is used to observe CDs, revealing their essential features such as particle shape, distribution of sizes, estimated average dimension, and crystalline arrangement.^76^ TEM having a resolution of 0.1–0.2 nm provides significant orders of amplification, thus helping in identifying the internal structure of the samples.^72^ Additionally, using high-resolution transmission electron microscopy (HRTEM), fine-structure measurements of CDs and spacing of lattices can be analyzed and compared with reference samples (graphitic carbon).^75^ Apart from some basic structural characterization carried out through HRTEM, the optical characterization is mainly focused on Fourier transform infrared (FTIR) spectroscopy, X-ray photoelectron spectroscopy (XPS), absorbance, and PL. The surface characteristics of the CDs are investigated using XPS and FTIR spectroscopy. The XPS identifies the elemental composition such as carbon, oxygen, hydrogen, nitrogen, and sulfur as well as their corresponding chemical state along with the overall electronic structure and the density of states. The FTIR reveals the presence of functional groups like hydroxyl (-OH), carboxyl (-COOH), or carbonyl (C=O) groups, which are abundantly present on CDs’ surface.^77^^,^^78^ These can be identified by the stretching and/or bending vibrations at distinct wavenumbers corresponding to the different groups.^72^^,^^79^

### Optical absorption

CDs exhibit varied absorption behaviors depending on their method of synthesis and the carbon sources.^87^ Normally, CDs are found to display strong absorption in the ultraviolet (UV) region, which extends into the visible region. The absorption peak of UV located in the range of 230–270 nm is due to the π-π∗ transitions of C-C bonds of the carbon core, and the absorption at wavelength range 300–340 nm is due to the n-π∗ transition of C=O surface groups.^79^^,^^88^ Further, CDs with sp^2^ domains (confirming the presence of π-conjugated electrons) along with (or without) linked surface groups/polymer chains, displayed absorption in the long wavelength range of 500–800 nm^89^^,^^90^^,^^91^ and emission in the red or near infrared (NIR) region. Thus, the absorption characteristics of CDs are a function of the π-conjugated domain size, the amount of heteroatom doping (nitrogen and oxygen) in the carbon core as well as the content/types of surface groups.

### Photoluminescence

**Figure 3:**
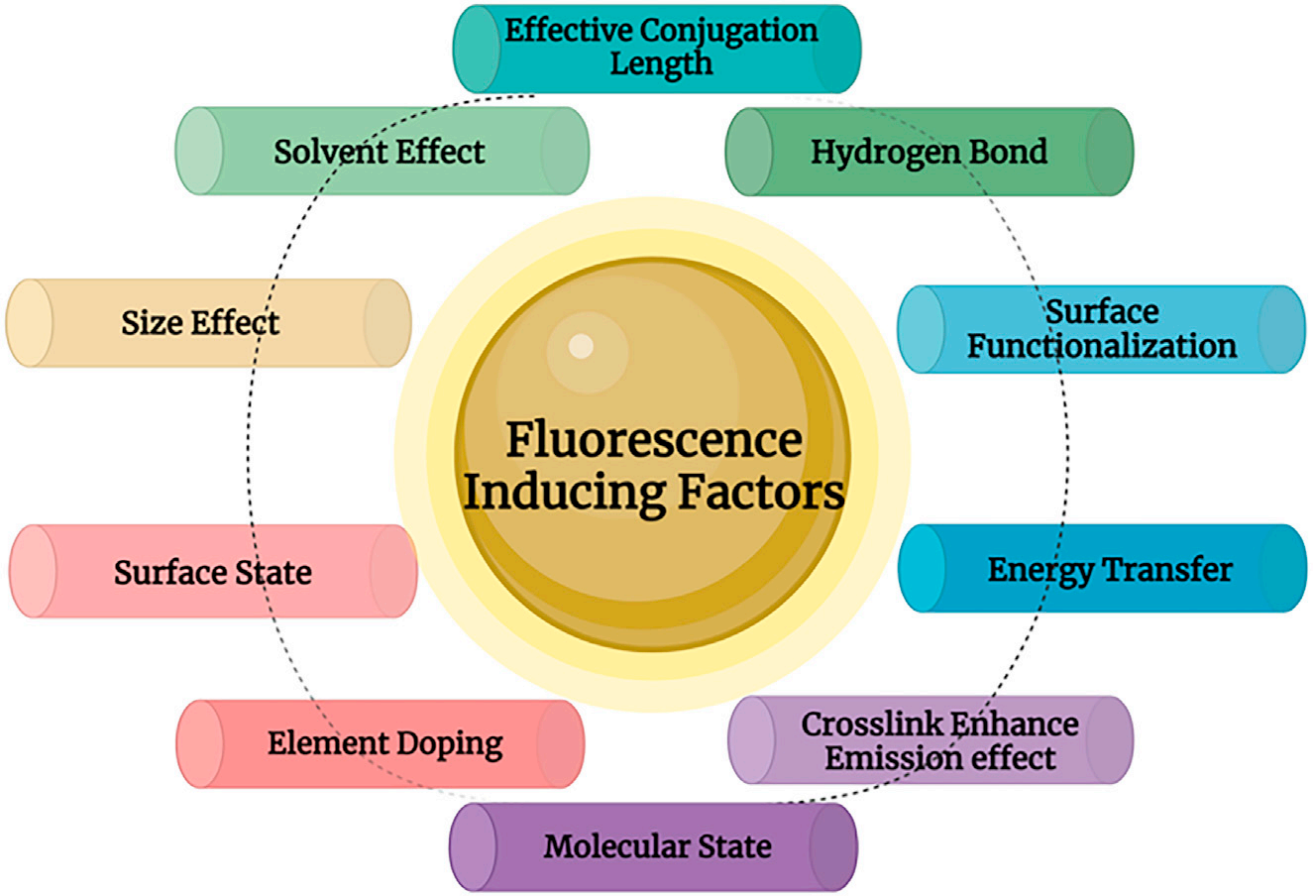
Different factors influencing photoluminescence in carbon dots

Due to their intense photoluminescence, CDs have gained interest as potential material for different analyte monitoring. The functionalization of CDs doped with heteroatoms, like nitrogen (N), phosphorous (P), boron (B), and sulfur (S), enhances their fluorescence characteristics and usage as a sensor.^27^ Apart from different synthetic precursors, size, edge geometry, hydrogen bonding, structural characteristics, passivation agents, defects,^92^^,^^93^ a variety of external conditions, such as temperature, pressure, solvent, and pH also affect the optical response of CDs (Figure 3). Hence, it is impossible to develop a single theory explaining the source of fluorescence^77^ and the origin of excitation-dependent PL. There are four types of mechanisms that have been largely followed by researchers: the quantum confinement effect, effective conjugation length, functional groups,^94^ and molecular state.

**The quantum confinement effect** (size effect/core emission) has been supported through numerous works.^77^^,^^94^^,^^95^ It is the transformation of quasi-continuous electronic energy levels near the Fermi-level into distinct energy levels on reduction of the particle size to the nanometer (nm) scale, i.e., the energy level splits or the energy gap widens. As a result of the carbon core’s sp^2^-conjugated domain, CDs display particle-size-dependent PL activity whenever dimensions become lesser than radius of their exciton.^96^ The π–π∗ transitions of sp^2^ clusters result in the radiative excitons recombination inside the core, assisted by quantum confinement effect, thus influencing the PL of CDs.^77^^,^^97^ However, the emission at core happens normally at shorter wavelengths, which exhibit a small QY. On the other hand, **the effective conjugate length** is the size of the sp^2^ (graphene)-conjugated domain and is smaller as compared with the actual particle size.^94^ In CDs, as the particle size increases, the bandgap is found to decrease, which further causes the PL emission wavelength to increase. Thus, the luminescence has a greater redshift effect with longer effective conjugate length.^98^ An enhanced interior graphene domain of CDs causes an increased electron delocalization and a uniform energy distribution, which further causes the energy gap between the Highest Occupied Molecular Orbital (HOMO) and Lowest Unoccupied Molecular Orbital (LUMO) to decrease.^99^ Another explanation of PL from CDs has been given through **surface passivation with functional groups (Surface States)**. The proposal that surface states may be used to control the PL in CDs was first given by Sun et al., by passivating the as-prepared CDs using polymer, diamine-terminated oligomeric poly-(ethylene glycol) (PEG_1500N_), converting them into bright luminescence-emitting CDs.^100^^,^^101^ It was suggested that specific functional groups (e.g., carboxyl, amine, and hydroxyl) were added to the surface of CDs, which have many energy levels between π and π∗ states,^102^ leading to stabilization of surface emissive traps.^94^ On illumination of CDs with a particular excitation wavelength, the observed emission is influenced by these emissive traps. More surface defects can result from a larger level of surface oxidation, leading the emission to red shift. Consequently, surface modification or passivation with various functional groups, particularly surface reduction and passivation of CDs, can effectively eliminate their non-radiative electron-hole recombination centers.^103^ This causes the QY to enhance and in turn tune their optical characteristics.^104^ Hence, manipulating the surface states (defect states) of CDs allows easy control of their PL properties. However, quantum confinement of emissive energy traps to the particle surface causes a large surface-to-volume ratio, which further displays strong PL upon surface passivation to exhibit strong photoluminescence.^101^ The **Molecular state theory** deals with the fluorescent molecular species (also referred to as molecular fluorophores), and by-products generated^100^ during the preparation of CDs (either free or bound to the CD surface) control the emission of CDs. Various groups have indicated that for the CDs synthesized using bottom-up approach, the fluorescent impurities determine the PL emission.^105^ As evidence to this theory, CDs synthesized using citric acid, revealed blue and green emission with usage of IPCA (imidazo [1,2-a] pyridine-7-carboxylic acid) and (HPPT) 4-hydroxy-1H-pyrrolo [3,4-c] pyridine-1,3,6 (2H,5H)-trione organic molecules, respectively.^77^

## Dye degradation using chemical/green-precursor-derived CDs

More than 10,000 variations of dyes are being utilized in textiles, leather, detergents, drugs, and cosmetics industries, with a yearly production of over 7 × 10^5^ tons dyes.^106^^,^^107^^,^^108^ The division of dyes is dependent on the nature of the source (natural or synthetic), chemical composition (chromophoric, anthraquinone, and acridine dyes), and their area of usage (dispersive dyes, azoic colors, and vat dyes). They can further be categorized as nonionic, anionic, and cationic dyes. The azo dyes are organic compounds with an azo group (-N=N-) and have industrial use in the textile, leather, and food sectors. Based on their charge, these dyes have been classified as either cationic or anionic azo dyes. The main cationic dyes are (1) **Methylene Blue (MB),** an organic thiazine cationic dye, a coloring agent used in the wood, cotton, and silk industries with adverse effects like mutations, skin irritations, eye burns, cancer, and gastrointestinal tract in humans^1^^,^^6^; (2) **Rhodamine B (RhB)**, an organic cationic dye, also extensively used in cosmetic, paper, and biochemical analyses^53^; (3) **Crystal Violet (CV)**, another organic cationic dye, generally employed as a purple color, in textile, paint, and printing industries can cause irritation to the gastrointestinal tract, eyes, and respiratory system.^109^ Further, its environmental degradation is complex due to its high organic structural complexity. (4) **Eosin Yellow (EY)** is a common cationic dye used in the textile industry for staining. It is carcinogenic and causes severe eyes and skin irritation.^110^^,^^111^ This dye is highly light sensitive, making its degradation difficult.^112^ (5) **Malachite Green (MG)** dye, a natural cationic dye, is widely used in fish farming, leather, silk, and wool industries.^113^^,^^114^ As this dye causes genetic harm, by entering the environment through sewage treatment units,^3^ it is restricted in several nations.^115^

The main anionic azo dyes are as follows: (1) **Naphthol Blue Black** (NBB) with acute environmental toxicity due to aniline, phenolic, sulfonated, and naphthalene groups, is commonly used in the textile industry for dyeing nylon, silk, wool, and textile printing.^116^ (2) **Metanil Yellow (MY**) and (3) **Methyl Orange (MO)** are well known for their usage as pH indicators and in the food sector. Despite identified applications, these dyes pose a severe hazard to the environment and human health, such as severe impact on the liver, DNA, and skin.^9^ **MO** (organic synthetic anionic azo dye) produces bright orange color on dissolving in water due to its very high colorability. It is highly teratogenic, toxic, carcinogenic, and harmful to the environment and living organisms.^117^ (4) **Congo Red (CR)** (synthetic anionic azo dye) is used in the leather, textile, paper, and plastic industries. Azo dyes are non-biodegradable and can enter the human body through the food chain. Further, this dye decomposes into aromatic amines and causes cancer in organs and tissues.^74^ (5) **Ponceau-4R (PR)** and **Allura Red (AR)** (synthetic anionic azo dyes) are water-soluble, red-colored dyes mainly used in foodstuffs like jelly, sweets, fruit juices, and drinks, especially in India. The overconsumption of these dyes leads to physical and mental effects and hyperactivity in children. It is also a reason for asthma, insomnia, headaches, and allergies in adults.^118^^,^^119^ As per European Food Safety Authority (EFSA), Allura Red and Ponceau-4R dyes’ safety limits are 0–7 mg/kg/bw/day and 0.025–0.5 g/kg, respectively.^120^

As part of the innovative sustainable methods, photocatalytic degradation of the above-mentioned dyes has been successfully achieved using CDs (and their composites) derived from some chemical and natural sources (Tables 1 and 2).

**Table 1:** Chemical-precursor-derived CDs and their composites: synthesis conditions and application as photocatalysts for dye degradation

S.No.	Precursor/Catalyst/Ref	Dye	Synthesis Technique	Average dimension of CDs (nm)/ Composite nanostructure	Light source	Rate constant (min^−1^)	Degradation (%)/Degradation time (min)	Parameters studied for Dye degradation	Recyclability (Degradation% achieved after nth cycle)
1	CTi-MS-15-X; (citric acid + N-(β-aminoethyl)- γ -aminopropyl methyl dimethoxy silane (AEAPMS) X = TEOS/TBOT^a^ (C. Cheng et al., 2016)^121^	Acid Orange 7 (azo dye)	One-pot co-condensation	9.33 nm/Mesoporous composite	300 W Xe lamp (UV to NIR) (λ ∼200–800 nm)	∼0.009	100/90	Effect of dopant N	–
2	CuS@CQDs@CHNS^b^ (glucose, CuSO_4_·5H_2_O + Na_2_S_2_O_3_·5H_2_O) (De et al., 2017)^122^	Rhodamine B	Hydrothermal	∼4 nm/Hollow nanosphere (HNS)	Sunlight	∼0.98	100/50	–	95%/5th cycle
3	B-doped CDs Citric acid (CA) +1,2-dibromoethane (DBE). (Peng et al., 2020)^123^	(a) Rhodamine B (b) Methylene Blue	Hydrothermal	4.7 nm/CDs	310 W Hg (Xe) lamp (visible light)	1.8 × 10^−2^ (RhB) 2.4 × 10^−2^ (MB)	∼100/107	Effect of dopant B	–
4	NCQDs/TiO_2_ (CCl_4_ and NaNH_2_) (Y.-Q. Zhang et al., 2013)^124^	Rhodamine B	Hydrothermal	20 nm/NCDs	500 W Xe lamp Solar light (λ > 400 nm)	0.11	>95/30	Effect of dopant N	–
5	NSP-CDs (Polyethylene glycol MW 400 (PEG-400) as a carbon source + phosphoric acid (H_3_PO_4_) as a phosphorus (P) dopant+ L-cysteine (Cys) as nitrogen (N)+ sulfur (S) dopants (Saini et al., 2021)^9^	(a) Metanil Yellow (b) Methyl Orange	Microwave	10 nm/CDs	Sunlight	MY-Bulb: 0.47 × 10^−2^; MO-Bulb: 0.29 × 10^−2^; MY-Sun: 3.71 × 10^−2^; MO-Sun: 0.47 × 10^−2^	∼20 ppm/∼60 (MY) and ∼90 (MO)	1. Effect of dopant N, S, P 2. Light source 3. pH 4. Trapping experiments (role of scavengers)	40%/5th cycle
6	N-CDs (Glucose + ammonium hydroxide) (Z. Ma et al., 2012)^125^	Methyl Orange	Ultrasonic	10 nm/CDs	150 W Xe lamp (Visible light)	–	90/120	Effect of dopant N	
7	CQDs/TiO_2_ Nanosheet composites (TNS) using graphite rods as both anode and cathode and NaOH/EtOH as electrolyte (Yu et al., 2014)^51^	Rhodamine B	Electrochemical	10 nm/TNS CQDs/TiO_2_ Nanosheet composite	500 W Xe lamp (Visible light)	–	95/60	1. Pure TNS 2. P25 (form of TiO_2_) 3. CQDs 4. CQDs/TNS 5. CQDs/P25 composites	
8	CQD/N-ZnO composites carbon black pigment (HIBLACK Orion) + ammonia + zinc acetate dihydrate (Muthulingam et al., 2015)^54^	(a) Malachite Green (b) Methylene Blue (c) Fluorescein dyes	Chemical method	∼2.5 nm/CQDs	Daylight	–	MG dye: 100/30 MB: 100/45 FL: 95/15	Effect of dopant N	In the 4th cycle, CQD/N-ZnO required 90, 65, and 85 min for complete degradation of MG, MB, and FL dyes
9	CQDs@CuS (ammonium citrate + ethylenediamine as carbon and nitrogen sources) (X. Wang et al., 2018)^39^	Methylene Blue	Hydrothermal	4 nm/CDs	500 W Xe lamp (Visible light)	–	99/40	1. pH (5, 7, 9, 11) 2. H_2_O_2_ 3. Irradiation time	92%/5th cycle
10	Magnetic CDs (glucose + Fe_3_O_4_) (Sun, 2018)^56^	Methylene Blue	–	5–10 nm/CDs	400 W Xe lamp (λ > 420 nm) (Visible light)	–	83/30	1. Temperature 2. Irradiation time	More than 10 cycles
11	N-GQDs/TiO_2_ (citric acid + ethylene diamine) (Safardoust-Hojaghan & Salavati-Niasari, 2017)^126^	Methylene Blue	Hydrothermal	(a) 14.8 nm for CDs (b) 65 nm for N-GQDs/TiO_2_ nanocomposite	400 W UV tube light		85/17	Irradiation time on 1. TiO_2_ 2. GQD-TiO_2_ composite	–
12	CDs (aqua mesophase pitch (AMP) N-CQDs (CQDs + ammonia) Cl-CQDs (CQDs + thionyl chloride) (Y. Cheng et al., 2019)^127^	(a) Rhodamine B (b) Methylene Blue (c) Indigo Carmine	Hydrothermal	(a) 2.8 nm (CQDs) (b) 4.5 nm (N-CQDs) (c) 4.2 nm(Cl-CQDs) (QY—27.6%)	Natural light	2.5 × 10^−2^ (N-CQDs, RhB)	1. For N-CQDs (a) 97/240 (RhB) (b) 23/240 (MB) (c) 56/240 (IC) 2. For Cl-CQDs (a) 25/240 (RhB) (b) 54/240 (MB) (c) 60/240 (IC)	1. Irradiation time 2. Effect of dopant Cl, N	93% in 5th cycle
13	Multi-layered GQDs (graphene oxide) (Umrao et al., 2015)^57^	(a) Methylene Blue monomer (MB+) (b) Methylene Blue dimer (MB^+^)_2_	Hydrothermal	∼8 nm/GQDs	1 Wmm^−2^ LEDs (λ = 470 and 520 nm)	(a) 0.056 (green light) 0.054 (blue light) (b) 0.024 (green light) 0.026 (blue light)	(a) 93.3/60 (GL) 89.4/60 (BL) (b) 78.8/60 (GL) 79.5/60 (BL)	1. Variation of light sources (blue [BL] and green [GL] LEDs) 2. Irradiation time	–
14	CDs (citric acid + 1,2-phenylenediamine) (Zhou et al., 2019)^66^	(a) Rhodamine B (b) Methylene Blue	Microwave	4, 3, 2 nm/CDs	UV lamp	(a) 1.3 × 10^−2^ (b) 3.6 × 10^−2^	(a) 50/60 (b) 90/60	1. Influence of size 2. Various charge-carrier scavengers	No change in degradation% after 4th cycle
15	CQDs/ZnO (graphite powder + Zn(NO_3_)_2_·6H_2_O) (Li et al., 2013)^128^	Rhodamine B	Sol-gel + spin coating	∼2–5 nm/CQDs	18 W UV lamp (UV lamp and visible light) (λ = 365–600 nm)	–	80/120	1. Pure ZnO 2. ZnO/CQDs (with different layers of CQD)	–
16	CQDs/ZnO (sucrose + Zn(NO_3_)_2_·6H_2_O) (Ding et al., 2016)^55^	(a) Rhodamine B (b) Methyl Orange (c) Methylene Blue	Hydrothermal	150 nm/CQDs/ZnO Nanocomposite	250 W Xe (UV and visible light)	(a) 0.0092 (RhB) (b) 0.0031 (MO) (c) 0.0121 (MB)	–	1. CQD amount 2. Irradiation time 3. Different types of dyes (RhB/MO/MB)	–
17	B-/P-modified CDs (phosphoric acid, 1, 2 dibromoethane and citric acid) (Yadav et al., 2023)^68^	Methylene Blue	(a) Hydrothermal BPCD(H) (b) Microwave BPCD(M)	CDs	(a) 100 W UV-visible regions (λmax = 660 nm) (b) UV region (λmax = 293 and 260 nm)	(a) 0.00689 BPCD(H) (b) 0.00812 BPCD(M)	95/60 (at pH = 11; source distance = 5 cm; power 100 W)	1. pH (3–11) 2. Dopants 3. Irradiation time 4. Effect of reactor design parameters (source distance and source power)	–
18	N-doped CDs (diammonium citrate) (Abbasi et al., 2023)^129^	(a) Methylene Blue (b) Congo Red	Pyrolysis	1.57 nm/CDs	Visible light	(a) 0.063 (MB) (b) 0.256 (CR)	(a) 96%/55 (MB) (b) 98%/60 (CR)	pH (3–9)	=

a TEOS: tetraethyl orthosilicate; TBOT: tetra butyl titanate

b CHNS: carbon hollow nanospheres

**Table 2:** Green-precursor-derived CDs and their composites: synthesis conditions and application as photocatalysts for dye degradation

S.No.	Precursor/Catalyst/Ref	Dye	Synthesis Technique	Average dimension of CD (nm)/composite nanostructure	Light source	Rate constant (min^−1^)	Degradation (%)/Degradation time (min)	Parameters studied for dye degradation	Recyclabiliy (Degradation% achieved after nth cycle)
1	Pear juice (Das et al., 2019)^4^	Methylene Blue	Hydrothermal	3–6 nm/CDs	60 W Tungsten lamp (Visible light)	0.03889	99.5/130	1. Irradiation time 2. Different scavengers : (a) Na_2_EDTA for holes (b) t-BA for hydroxyl radicals (c) p-BZQ for superoxide radicals	
2	Rice bran CDs (Jothi et al., 2021)^20^	Methylene Blue	Hydrothermal	2.96 nm/CDs	400 W Xe lamp	–	89.20/20	Irradiation time	–
3	Pomelo juice and ammonium bicarbonate N-CQDs (Ramar et al., 2018)^14^	Methylene Blue	Hydrothermal	3 nm (Undoped) and 70 nm (N doped)	(1) 20 W compact fluorescent lamp (2) Sunlight	–	–	1. Dopants 2. Light sources as sunlight and compact fluorescent lamp 3. Irradiation time	–
4	Orange waste peels CDs/ZnO (Prasannan & Imae, 2013)^18^	Naphthol Blue-Black	Hydrothermal	2−7 nm/CDs	UV light	–	100/45	Irradiation time	–
5	Corn powder (a) GQDs (b) GQDs/TiO_2_ (Teymourinia et al., 2017)^53^	Rhodamine B	(a) Hydrothermal (b) *In Situ* sonochemistry method	(a) 20–40 nm (GQDs) (b) 20 nm and 60 nm (GQDs/TiO2)/nanocomposite	UV light	–	(a) 45/80 (b) 100/80	Irradiation time	–
6	Bass CDs Fish scales (Bass fish) (Campalani et al., 2021)^50^	Methyl Viologen	Hydrothermal	120 nm/CDs	80 W UV	–	62.5/30	1. Irradiation time 2. Precursors 3. Synthesis technique	–
7	Aloe barbadensis miller (aloe vera) (Malavika et al., 2021)^21^	Eosin Yellow	Microwave-assisted reflux synthesis	<5 nm/CDs	Sunlight	2.079×10^–2^	98.55/80 and 100/100	Irradiation time	–
8	Elettaria cardamomum (Zaib et al., 2021)^130^	(a) Congo Red (b) Methylene Blue	Ultrasonication	-/Amorphous	100 W Tungsten bulb	(a) (146–19)×10^−4^/min for 5–75 ppm (b) (88–15)×10^−4^/min for 5–75 ppm	–	1. Dye concentration 2. Different types of dyes 3. pH 4. Volume of CDs 5. Irradiation time	–
9	Empty fruit bunches (oil palm) + sodium thiosulfate pentahydrate) (Abd Rani et al., 2021)^5^	Crystal Violet	Hydrothermal	2.9 nm/CDs	100 W UV light	–	99.7/200	1. pH 2. Irradiation time 3. Temperature (also analyzed through theoretical approach)	70/5th cycle
10	Gum Ghatti, a natural exudate from tree Anogeissus latifolia CDs-ZnO (Sekar &Yadav, 2021)^3^	Malachite Green	Microwave pyrolysis	2.6 nm/ CDs-ZnO nanocomposite	Visible light	0.01072	94.8/60	1. ZnO addition 2. Catalyst loading 3. Initial dye concentration 4. pH 5. Scavengers (ethanol, NaCl, ascorbic acid and the K_2_Cr_2_O_7_)	75/3rd cycle
11	Bougainvillea/r-Mg-N-CDs (Bhati et al., 2018)^67^	Methylene Blue	Microwave	16–18 nm/CDs	(a) Sunlight (b) Tungsten bulb (100 W)	–	(a) 99.1/120 (b) 45/120	Scavengers: para- benzoquinone for superoxide (O_2_) DisodiumEthylenediamin etetraacetate (Na_2_-EDTA) for holes, tertiary butyl alcohol (t-BA) for hydroxyl	∼62/4th cycle
12	Instant coffee CDs/ZnO (Omer et al., 2018)^52^	Methylene Blue	Hydrothermal	3–4 nm/CDs	(a) 15 W cm^−2^ weak LED light (visible-NIR region) (b) 15 W UV Hg lamp (λ = 365 nm)	(a) 0.140/60 (Visible-NIR region) (b) 0.0139 (UV)	(a) 80/600 (b) 75/100	Different light sources (excitation wavelength)	–
13	Lemon peel waste CQDs/TiO_2_ (Tyagi et al., 2016)^46^	Methylene Blue	Hydrothermal	1–3 nm/CDs	12 W UV	0.0136	–	–	–
14	Peach fruit juice ZnO@N-CDs (Atchudan, Edison, Perumal, Karthik et al., 2018)^7^	Methylene Blue	Hydrothermal	3–5 nm/N CDs	UV	0.0456	>95%/60	Irradiation time	–
15	Peach fruit juice TiO_2_/N-CDs (Aqueous ammonia as N source) (Atchudan, Edison, Perumal, Vinodh et al., 2018)^6^	Methylene Blue	Hydrothermal	5.2 nm/nanocomposite	UV	0.0593	90/40	Irradiation time	3 times
16	Turmeric powder CQDs/CoFe_2_O_4_ (Ahmadian-Fard-Fini et al., 2017)^37^	Acid Azo dyes (a) Acid Black: 24, (b) Acid Brown: 14, (c) Acid Red: 1	Hydrothermal	20–100 nm/CDs	8 W UV lamp	–	(a) 95/60 (b) 90/90 (c) 65/120	1. Irradiation time 2. Different dyes	–
17	F/N-CQDs (Gourd flesh powder as C and F source)/tricolor F/N-X-CDS (X = G [green], Y [yellow], O [orange]) + Urea (N source) (C. Wang et al., 2021)^1^	(a) Methylene Blue (b) Congo Red	Hydrothermal	∼6.07 nm (G) ∼4.58 nm (Y) ∼5.09 nm (O)	UV	(a) MB 0.0432 (G), 0.0566 (Y), 0.0678 (O) (b) CR 0.2059 (G), 0.2706 (Y), 0.4095 (O)	(a) MB 100/60 (G) 100/70 (Y) 100/90 (O) (b) CR 98/10 (G) 98/15 (Y) 98/20 (O)	1. Dark and sunlight irradiation 2. Irradiation time 3. Different scavengers- benzoquinone, dimethyl sulfoxide (DMSO), Na_2_-EDTA	–
18	Bitter apple peels CDs (Aggarwal et al., 2020)^33^	Crystal Violet	Carbonization	–	Sunlight	–	∼100/∼ 90	1. Dye concentration 2. Catalyst loading	∼50 in 6th cycle
19	Grass, CQDs (Sabet & Mahdavi, 2019)^36^	(a) Acid Blue (b) Acid Red (c) Eosin Yellow (d) Methylene Blue (e) Eriochrome Black T (f) Methylene Orange	Hydrothermal	<10 nm	(1) UV (2) Visible	–	(a) 100/30 (b) 100/30 (c) 100/90 (d) 100/90 (e) 100 (without any radiation)	1. Catalyst loading 2. pH 3. Irradiation time 3. Light source	–
20	(a) N-CDs (Olive pit biomass liquor derived) (b) N-CDs (commercial xylose)/ (VOPO_4_ and NbOPO_4_) (Carballo et al., 2023)^131^	Methyl Orange	Hydrothermal	(a) 15 nm/CDs (b) 2–6 nm/CDs	UV	–	(a) 0.05 ppm/80 (b) > 0.03 ppm/80	–	–

Tables 1 and 2 indicate the preferential degradation of **MB** dye relative to other dyes, with the maximum photocatalytic activity reported for CDs-based composites with semiconductor oxides as compared with doped and undoped CDs. CQDs@CuS nanocomposite, due to lower band gap energy, revealed increased photocatalytic behavior for **MB** dye because of the electronic interaction of CuS with CQDs.^39^ On introducing Fe_3_O_4_ nanoparticles to CQDs, photocatalytic activity is enhanced by more than 10 times as compared with Fe_3_O_4_ nanoparticles alone.^56^ On adding N-GQDs into TiO_2_ nanoparticles, the photodegradation ability of TiO_2_ nanoparticles toward **MB** increases from 40% to 85%.^126^ The degradation of **MB** dye using multi-layered GQDs into non-toxic environment products was reported, with higher degradation efficiency for monomer **MB**^**+**^ compared with dimer (**MB**^**+**^**)_2_**. It is due to the extraction of a proton from GQDs leading to the development of leucomethylene blue (LMB), which is weakly bound to GQDs with hydrogen bonds.^57^

As a particular example, the red-emitting magnesium-nitrogen-embedded carbon dots (r-Mg-N-CDs) showed about six times increased photodegradation under sunlight, compared with the tungsten bulb (100 W).^67^ The rate constant of ZnO-CDs (0.14/h) is reported to be 23 times more significant than ZnO (0.006/h), whereas the rate constant of ZnO alone is 14 times more than CDs (0.01/h) using a weak LED lamp. Similar behavior was reflected using UV source also.^52^ The lemon-peel-derived water-soluble CQDs selectively detected Cr (VI) with a limit of detection as 73 nM and QY 9% after 1-year storage in contrast to 14% of fresh samples. Further, TiO_2_–CQDs composite was used to degrade **MB** dye with 2.5 times higher efficiency than TiO_2_ nanofibers, due to increased charge separation at the contact.^46^ The TiO_2_ NPs@CDs nanocomposite (peach fruit extract as precursor) revealed five times increased photocatalytic degradation efficiency of **MB** dye compared with TiO_2_ NPs.^6^

The degradation efficiency of CDs-modified porous ZnO nanorods was 2.5 times higher than the porous ZnO nanorods. The heterostructure CDs/ZnO also display the same behavior with four-layered CQDs heterostructure with further enhancement in degradation efficiency.^128^ GQDs (using corn powder)/TiO_2_ nanocomposite revealed more significant photocatalytic activity than GQD and TiO_2_ nanoparticles due to the relatively low recombination rate of photogenerated electron-hole pairs in the nanocomposite.^53^

In sulfur-doped CDs prepared using empty fruit bunches (a by-product of the Palm Oil Mill Industry as carbon precursor and sodium thiosulfate pentahydrate), a good photocatalytic behavior toward **CV** has been attributed to the small size of CQDs.^5^ The degradation efficiency of azo dye **NBB** with CQDs (using orange waste peels), ZnO, and CQDs/ZnO composite was observed as 4.4%, 84.3%, and 100% in 45 min, respectively, revealing the effectiveness of composite over ZnO and CQDs alone. It is attributed to the CDs' large surface area and electronic charge interactions.^18^ Organic anionic Azo dyes (acid black 24, acid brown 14, and acid red 1) decomposed to water, carbon dioxide, and residuals in the presence of cobalt ferrite (CoFe_2_O_4_—carbon nanocomposite) using turmeric as precursor.^37^ CQDs synthesized using grass were able to degrade six different dyes, **Acid Blue, Acid Red, EY, Eriochrome Black T, MO**, including **MB**.^36^ Zaib et al.^130^ revealed (*Elettaria-cardamomum*-derived CDs) that the kinetic rate constant of **CR** dye is more than **MB** due to the two azo groups linked to the **CR** dye.

## Main parameters influencing the photocatalytic activity

**Figure 4:**
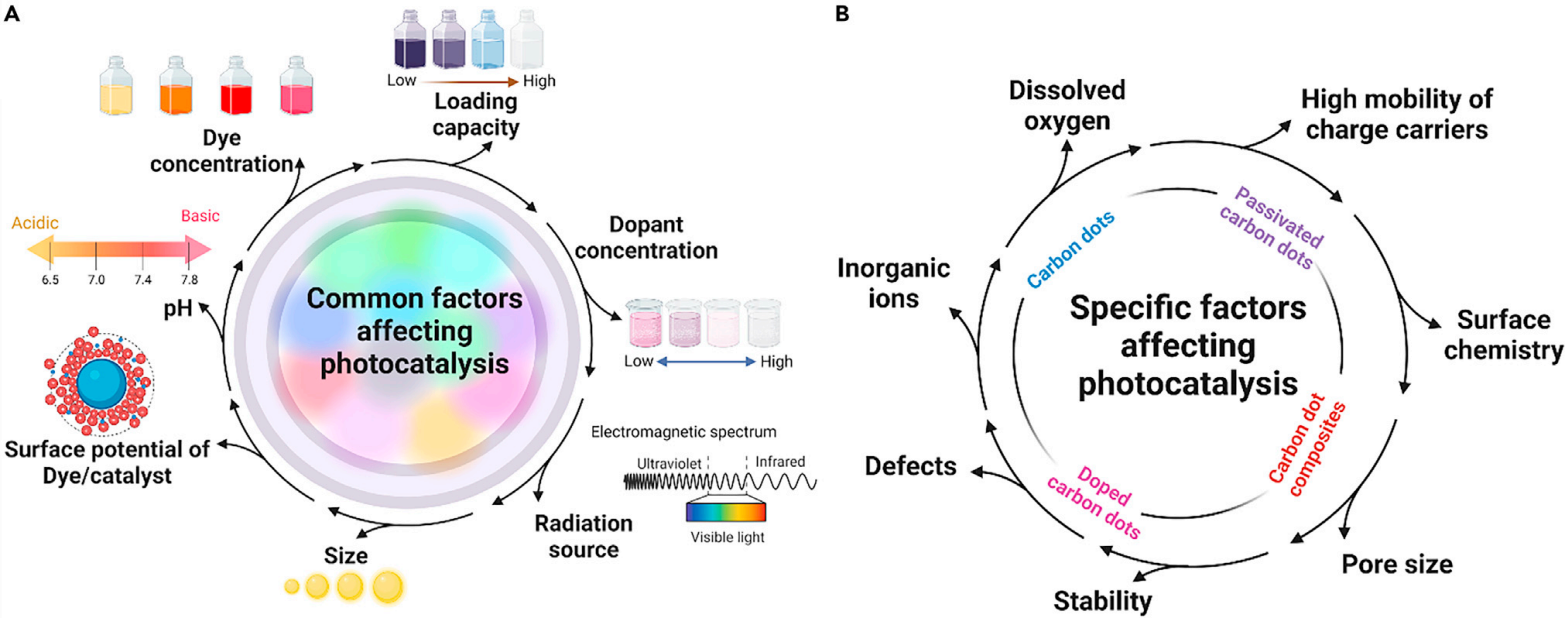
Factors affecting the photocatalytic efficiency (A) Common and (B) specific factors affecting the photocatalytic efficiency of CDs and their composites/hybrids.

In addition to various common parameters such as pH, dopants, temperature, light intensity, irradiation time, and photocatalysts/dye concentration that are primarily responsible for influencing the photocatalytic efficiency (Figure 4A), the surface functionalization, passivation, and scavengers are also discussed in this section.

### pH

The pH of the solution is a crucial factor in photocatalytic activity, i.e., excessive acid or base will affect the rate of degradation^132^ of the anionic or cationic dyes. An alkaline medium will help adsorption of cationic dye, whereas an acidic medium is responsible for anionic dye adsorption on the CD’s surface.^29^ The pH value can influence the photocatalytic phenomena in three different stages, namely during synthesis of catalysts, overall reaction mechanism, and the direct impact of the catalyzed component. The catalyzed material’s charge and shape are mostly governed by the pH of the solution.^24^^,^^29^ For example, the nitrogen-, sulfur-, and phosphorus-doped CDs (NSP-CDs) revealed the highest degradation efficiency (>98.78%) at pH 4, for **MY** and **MO** dyes.^9^ Another work from Wang et al. group revealed that **MB** degradation increased with the increase in the value of pH in the range 5–11.^133^ In boron- and phosphorus-doped CDs,^68^ removal efficiency of **MB** dye has been reported to increase from 32% to 94% with increase in pH from 3 to 11. In *Gum-Ghatti*-derived CDs, 94% degradation of **MG** dye was achieved at pH 8 and minimum efficiency (34%) at pH 3 for the degradation of **MG**.^3^ Zaib et al.^130^ revealed (*Elettaria-cardamomum*-derived CDs) that **MB** degrades more rapidly in an alkaline medium (pH 8).

### Concentration of the dye

The percentage of dye adsorption on the photocatalyst’s surface is the key factor in degradation of dye, using a photocatalytic mechanism perspective. As the mechanism is dependent on the initial amount of the dye, the rate of degradation declines as the dye concentration rises, due to aggregation of dye that decreases the light transmittance and scarce availability of active sites, leading to poor adsorption on the catalytic surface.^24^^,^^29^ Sekar (2021) et al.^3^ studied the effect of introducing different concentrations of the **MG** dye, 0.04–0.08 g/L, on the degradation efficiency, and revealed the maximum decolorization at 0.05 g/L at pH 8. Similarly, photodegradation rate was decreased with increasing **CV** dye concentration from 40 ppm to 100 ppm, under sunlight irradiation in 90 min in bitter-apple-peel-derived CDs.^33^

### Concentration of the dopant/composite

Dye degradation using photocatalysis is enhanced with an increase in the concentration of dopant due to availability of more light-generated EHP (electron-hole pair) recombination centers and creation of vacancies, to capture more oxygen.^29^ Peng et al.^134^ reported that boron doping in CDs increased **MB** and **RhB** dye degradation efficiency by 27% and 64%, in comparison to pure CDs. In addition, the photocatalytic activity of doped CDs is also increased by making composites with semiconductor oxide (TiO_2_, ZnO).^121^^,^^135^ In NCQDs/TiO_2_, photocatalytic activity is enhanced as compared with CQD/TiO_2_.^56^^,^^124^ Also, the degradation of **MB** dye with CQDs@CuS nanocomposite (10% degradation efficiency/40 min) is remarkably enhanced by incorporating H_2_O_2_ (100% degradation efficiency/40 min).^39^

### Surface potential of the dye/photocatalyst

The CDs modifications for photocatalytic usage can be achieved by using element doping, composite synthesis, surface passivation, and functionalization.^136^ The redox reactions kinematics can be controlled using the photocatalysts' surface functionalization, which is used to separate holes and electrons and prevent recombination of electron-hole pairs, by trapping the photo-generated electrons in several surface sites. Further surface passivation allows increase in photostability of catalysts and can be achieved with the usage of passivating agents such as poly(ethylene glycol) diamine.^136^

### Dosage of the photocatalyst/loading capacity

Although, an increase in the concentration of the photocatalyst causes an increase in surface area and active sites for dye adsorption, leading to an increase in reaction rate. However, after a certain optimum value of the photocatalyst, the number of free electrons ceases to increase further, and the reaction rate decreases. In *Elettaria-cardamomum*-derived CDs, the maximum **CR** and **MB** dyes degradation rate was achieved at volume of 5 mL.^130^ In bitter apple peel synthesized CDs, degradation rate to remove **CV** dye was enhanced with photocatalyst loading.^33^

### Effect of source (pulsed vs. continuous) wavelength and intensity/irradiation time

One of the main factors affecting the activity of a photocatalyst is the nature of the light adsorbed. Irradiating light on the surface of the photocatalyst directly initiates the formation of hydroxyl radicals moderating the e^-^-h^+^ recombination and leading to an increase in the reaction rate.^29^^,^^137^ The course of the photodegradation reaction is independent of the chararcteristics of the light, or the process of band-gap sensitization.^138^ Generally, the reaction rate has linear relationship with light intensity for low levels (<20 mW cm^−2^), while at intermediate levels (∼25 mW cm^−2^), e^-^-h^+^ pairs are generated at a faster rate of the reaction with minimal separation leading to a square root dependence with light intensity. However, upon increasing the light intensity beyond a certain optimum level, the simultaneous generation and recombination of e^-^-h^+^pairs causes the reaction rate to be unaffected by the light intensity.^139^^,^^140^ For example, **MG** dye degradation with/without CDs/ZnO composite, under dark conditions did not show any activity, whereas the dye degradation without the composite under visible light showed 2% decolorization, which confirmed enhanced dye degradation with increase in light intensity.^3^ Furthermore, varying the wavelength of light causes the photocatalytic efficiency to vary. Saini et al.^9^ found that bulb light was insufficient to totally degrade both **MY** and **MO** dye. Additionally, the rate of photodegradation is 10–13 times higher in the presence of sunlight than it is with artificial light (100 W bulb). Additionally, Zhou et al.^66^ studied photocatalytic activity in the presence and absence of UV cut-off filter (λ < 420 nm) and showed that visible light drives the photocatalytic activity of the CDs. NSP-doped CDs exhibited improved photocatalytic phenomena toward the degradation of **MY** and **MO** dyes because of the increased light absorption, primarily due to the surface defects and dopants.

### Role of scavengers

The scavengers are employed in conducting trapping experiments to identify the vital species that control photocatalytic degradation.^29^ The commonly used scavengers include (1) sodium ethylenediaminetetraacetate dihydrate (Na_2_-EDTA) and ammonium oxalate (AO) (to trap h^+^); (2) para-benzoquinone (p-BZQ) and ascorbic acid (AA) (to trap superoxide anion radicals); and (3) tertiary butyl alcohol (t-BA), isopropyl alcohol (IPA), and ethanol (to trap hydroxyl radicals). In pear-juice-derived CDs, Na_2_EDTA, t-BA, and p-BZQ decreased the efficiency of degradation to ∼25%, ∼33%, and ∼50% respectively.^4^ The **MG** dye degradation was decreased to 53.85%, 69.23%, 16.24%, and 8.55%, on the usage of NaCl, ethanol, K_2_Cr_2_O_7_, and ascorbic acid, respectively, as compared with 94.8% without the addition of scavengers.^54^

### Size of the CDs

The small size of CDs is essential to have a large surface-to-volume ratio, which further results in a significant number of defect sites. Consequently, the bandgap of CDs and their capacity to absorb light are influenced, resulting in enhanced photocatalytic activity.^2^ The work from Zhou et al.^66^ revealed the dependence of photocatalytic activity on the CDs’ size, i.e., smaller CDs exhibited enhanced activity in the degradation of **RhB** and **MB** dyes. Similarly, Prasannan et al.^18^ in their studies with C-dots/ZnO composites while degrading **NBB** azo dye under UV light demonstrated that the smaller size of CDs led to a larger surface area, which in turn causes greater absorption of light and also provides more surface coordination sites during photocatalysis.

### Other factors

Apart from the above-mentioned factors, the photocatalytic performance depends on the surface morphology, pore size, volume, and surface area (Figure 4B). C. Cheng et al.^127^ revealed the effect of the abundant mesoporous structure of CD and Ti composite on the enhancement of photodegradation efficiency of **AO** (**Acid Orange**)7. It is often dispersed as nanomaterials (CDs) on a support material (metal oxides) to enhance the exploitation of photocatalytic materials. Subsequently, the photocatalytic performance is an accumulated effect of several number of factors, including some specific factors of the photocatalytic particles such as high mobility of charge carriers, surface chemistry, stability, defects, inorganic ions, dissolved oxygen, and the interaction between the photoactive particles and the support material.

## Theoretical tool to guide catalyst design

Understanding the light-harvesting ability of CDs requires a better knowledge of the fundamentals of electronic and energy bands.^29^ Extensive research has been done on the photocatalytic activity of CDs and their dopants/composites using density functional theory (DFT) simulations, which is a tool to guide catalyst design. To increase the photocatalytic activity in case of CD-based heterostructures, different material designs have been tested to adjust the relative band locations (alignment of the bands and the properties at the juncture) among the two components i.e., CDs and the semiconductor/metal oxides. Simulations using DFT revealed that CDs, in their capacity as electron reservoirs, possess a variable bandgap.^141^

The HOMO/LUMO levels of CDs are influenced by several variables such as their size and shape. However, precise control over many of these variables remains difficult, hence posing a challenge in the experimental adjustment of the band locations of CDs. In contrast, the band structures of semiconductors are adjustable.^141^ Various researchers have studied CDs combined with adjustable bandgap semiconductors, such as Fe_3_O_4_, nitrogen-doped ZnO, etc. to explore the photocatalytic capabilities of the resulting heterostructures. DFT simulations of CD-enhanced BiSbO_4_ composites (CDs-BiSbO_4_)^142^ confirm the existence of a substantial electron screening effect within the layers between CDs and BiSbO_4_, generating a high electron transfer tunnel at the interlayer. The addition of CDs can hasten the movement of photoinduced electrons, resulting in a greater number of electrons and holes being created on the surface of the catalyst. Simulations using DFT reveal that photoinduced electrons originate in O 2s orbitals (VB of BiSbO_4_), undergo excitation to Bi and Sb hybrid orbitals (CB of BiSbO_4_), and eventually arrive at CD’s C 2s orbitals.^142^ In multilayered graphene quantum dots (MLGQDs),^57^ the application of DFT has been utilized to gain insight into the interaction between MLGQDs and **MB** dye. The results derived from DFT simulations demonstrate that the monomer (**MB**) removes a proton from the hydroxyl group. This process leads to the creation of leucomethylene blue (**LMB**), which reveals a weak hydrogen bond with MLGQDs. Additionally, TD-DFT (time-dependent density functional theory) has also been used to determine the HOMO and LUMO of these charge transfer compounds.^57^

## Photocatalytic degradation mechanism of organic dyes

Mechanistic understanding of photocatalytic degradation of organic dyes using CDs and their composite material is still under debate. Researchers have concluded that CDs could be considered semiconductors that generate electron-hole (e^−^-h^+^) pairs under light irradiation.^96^ Usually, the band gaps of CDs are not dependent on the change in VB/CB energies; instead, the band gaps can be altered/tuned by the surface states, conjugation length, etc. Due to this reason, most of the CDs have been considered for photocatalytic redox reactions. Although CDs alone have exhibited low photocatalytic efficiency (due to low quantum yield and nonreusable nature), encouraging studies on modified/doped CDs and their composite systems have revealed effective photocatalytic dye degradation.

### Carbon dots as photocatalyst in H_2_O

**Figure 5:**
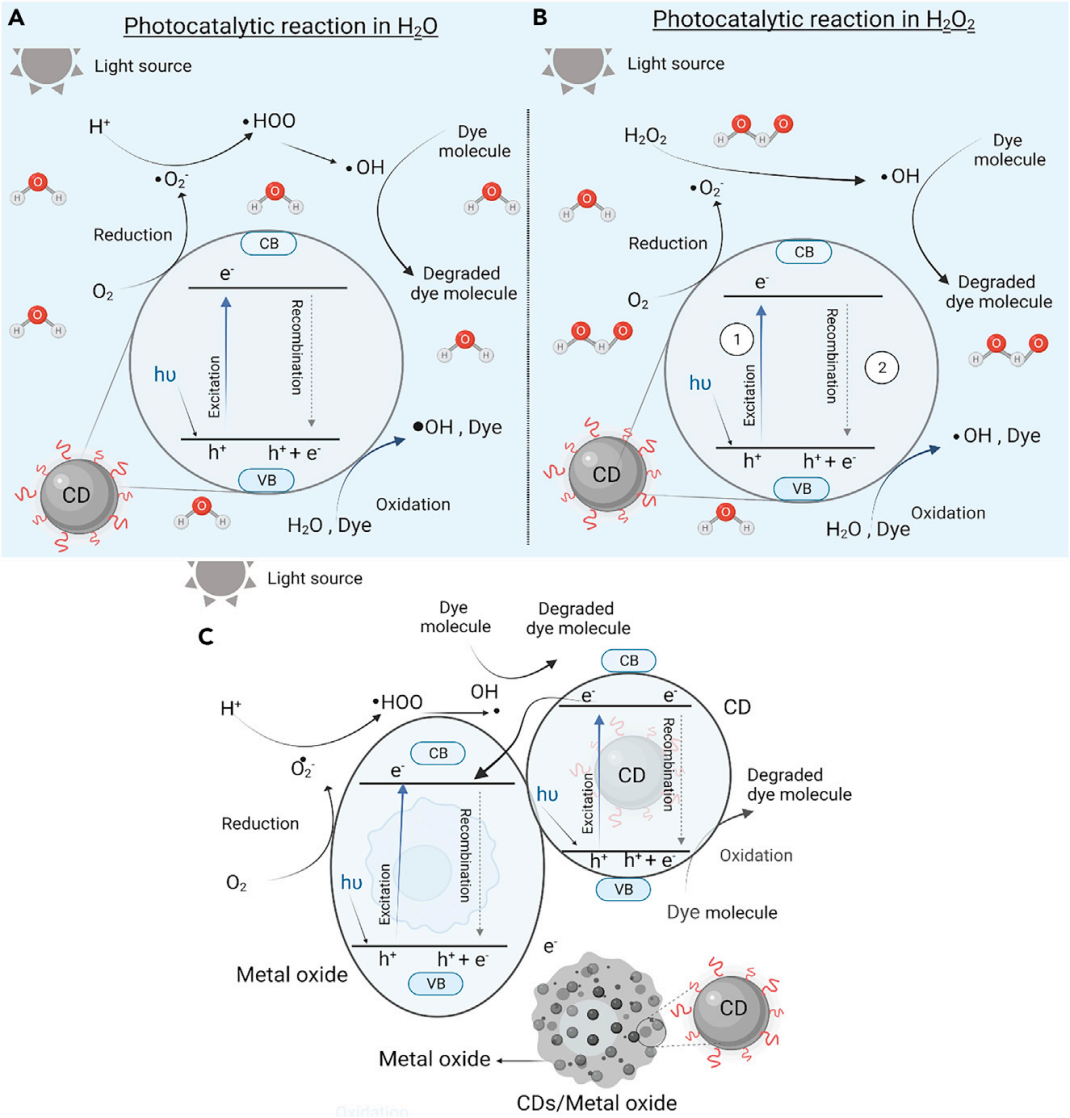
Different mechanisms Reported mechanisms for CDs and their composites-based photocatalytic activity in the presence of (A) H_2_O, (B) H_2_O + H_2_O_2_, and (C) CDs hybrid structures in H_2_O.

In general, degradation of the dyes using photocatalysis go through six different stages (Figure 5A): (1) the photoexcitation of CDs and generation of e^-^-h^+^ pair; (2) water molecule separation as hydrogen (H^+^) and hydroxyl (OH^−^) ions; (3) the generated electron (e^−^) contributes in the formation of reactive superoxide anion radical (·O_2_^−^) species using the adsorbed oxygen (O_2_); (4) the ·O_2_^−^ reacts with hydrogen ion (H^+^) and forms hydroperoxyl (hydrogen superoxide) radical HOO^·^; (5) hydroperoxyl radical (HOO^·^) results in the formation of hydrogen peroxide (H_2_O_2_); and (6) absorption of electron by hydrogen peroxide to form hydroxyl radical (·OH). As the generated ·OH radicals (reactive species) are powerful oxidizing agents, the resulting photocatalytic activity degrades the dye molecule. Alternately, the generated hole can oxidize the dye directly and is reflected in the form of its discoloration. The following equations summarize the photocatalytic degradation mechanism using CDs as photocatalyst:

(Equation 1) $$CDs+h\nu {\;\to \;e}^{-}{+h}^{+}$$

(Equation 2) $$H_{2}{O\;\to \;H}^{+}{+OH}^{-}$$

(Equation 3) $$e^{-}{+adsorbed\;O}_{2}\to \;\cdot {O_{2}}^{-}$$

(Equation 4a) $${{\cdot O}_{2}}^{-}{+H}^{+}\;\to \;\cdot HOO$$

(Equation 4b) $${2\;\cdot HOO\;\to \;H}_{2}O_{2}{+O}_{2}$$

(Equation 4c) $$H_{2}O_{2}{+e}^{-}{\;\to \;\cdot OH+OH}^{-}$$

(Equation 5) $${4\cdot OH+Dye\;\to \;degraded\;products+2H}_{2}{O+O}_{2}$$

or alternately

(Equation 6) $${\left(oxidation\right)\;Dye\;molecule+h}^{+}\;\to \;degraded\;products$$

In addition, as mentioned earlier, identification of the vital species (·O_2_^−^, ·OH, h^+^) that directly control the photocatalytic efficiencies has been done through trapping experiments. Saini et al.^9^ show that p-benzoquinone (p-BZQ) degradation is slower (minimum degradation took place due to the trapping of ·O_2_^−^) in comparison to the sodium salt of ethylenediamine (∼50% and ∼70% degradation for **MY** and **MO** dyes) and *tert*-butyl alcohol (∼60% and ∼80% degradation for **MY** and **MO** dyes) within 60 and 90 min for **MY** and **MO** dyes, respectively. Through these trapping results, the authors could conclude that ·O_2_^−^ species play a significant role by reacting with the adsorbed dye molecules on the catalyst’s surface. The radicals help in the decomposition of the larger molecules into smaller fragments. Similarly, the decreased photocatalytic activity results obtained after the removal of dissolved oxygen species in water^67^ by Argon confirmed its important role in the dye degradation. Additionally, the electrostatic adsorption of dyes onto CDs must be taken into consideration.

### Carbon dots as photocatalyst in presence of H_2_O_2_

The synergetic effect that results from combining CDs with H_2_O_2_ (experimentally shown by Wang et al.^39^), generates ·OH radicals.^143^^,^^144^ The possible steps that correspond to the cleavage of H_2_O_2_ are shown in the following equations (also summarized schematically in Figure 5B):

(Equation 7) $$e^{-}{+H}_{2}O_{2}{\;\to \;\cdot OH+OH}^{-}$$

(Equation 8) $${OH}^{-}{+h}^{+}\;\to \;\cdot OH$$

The degradation of the dye will take place in the similar way as mentioned earlier.

### Carbon dots with metal oxide as hybrid photocatalyst

Fast recombination of e^−^-h^+^ directly affects the activity of the free radicals,^145^ and hence low photocatalytic efficiency has been reported using bare CDs. Formation of hybrid structures with metal oxide/semiconductors (as suggested in examples in Tables 1 and 2) facilitates the transfer of electrons to the conduction band of the semiconductor as CDs can act as bridges for electron conduction. In addition, to increase in the effective surface area and more light absorption over the wider range of visible spectrum, CDs’ induced sensitizing phenomenon results in the reduction of the energy gap. The possible tuning of the electronic bandgap or specifically its position (in metal oxide/semiconductor) escalates the reduction/oxidation capability of the generated e^−^-h^+^ pair. Hence, apart from possible reusability, the composite structures have been successfully tested (Tables 1 and 2) to promote the segregation of e^−^ and h^+^ and in turn expedite the electron transfer.^146^ After the generation of the e^−^-h^+^ pair, the Equation 3 to Equation 6 (given earlier) can be followed for the mechanistic understanding of the photocatalytic dye degradation using CDs-metal oxide (CD-M) composites as well (Figure 5C).

As an example, Cheng et al.^121^ reported a synergy effect between CD and Ti in the silica matrix through enhanced light absorption capacity and increased catalytic efficiency. The enhancement in the light absorption was confirmed through a decrease in the PL signal intensity of CDs with Ti. In addition, the authors confirmed that the TiO_2_-CDs linkages can be attributed to generating ·O_2_^−^. In another study, the CDs-TiO_2_ in mesoporous silica were found to promote the charge transfer to the catalysts’ surface. Zhang et al.^124^ observed superior catalytic activity with N doping in CDs with TiO_2_ due to the lower work function of N-doped CDs than undoped CDs, and the photogenerated electrons easily transfer to the conduction band of TiO_2_. Similarly, CDs with ZnO structures possess a stronger electronic coupling of π electrons of carbon with the conduction band (CB) of ZnO due to the difference in CB energies of ZnO (−4.05 eV) and CDs (−4.5 eV).^54^ Furthermore, Yu et al.^51^ studied TiO_2_ facet-based catalytic activity, and the transient current responses of CDs/TiO_2_ nanosheets (TNS) ({001} facets) and CDs/P25 ({101} facets of anatase TiO_2_) composites was measured. The CDs/TNS composites exhibit three times higher transient photocurrent response than that of CDs/P25 composites. Due to the enhanced photoactivated e^−^-h^+^ pairs in CDs/P25 composites, the **RhB** degradation efficiency is higher through the CDs/TNS system.

## Conclusions and future perspectives

The presence of organic dyes in the contaminated aqueous system poses a significant threat to living organisms in sea/river water. Consequently, efforts are being made to reduce their usage, and enhance the investigation to monitor and minimize the contamination originated from the organic dyes in the environment. However, degradation of contaminated water from the environment remains challenging for scientists. For setting up a precise and accessible procedure to purify contaminated water, it is important to utilize various qualitative and quantitative systematic approaches, such as microscopic and spectroscopic methods, to recognize the type of photocatalyst and their efficiency toward the decontamination of the ecosystem. The combination of systematic procedures could facilitate an improvement in the photodegradation process. In this regard, sustainable materials such as CDs from green precursors are not just economical and easy to use but also good for enhanced photocatalytic performance if supported on metal oxides/semiconductor.

The **principal benefits of CDs** as potential photocatalyst for various dyes can be summarized as cost-effectiveness, ease of fabrication, excellent optical properties, biocompatibility, ease of passivation, and tunability of bandgap. The availability of multiple functional groups in green-precursor derived CDs results in enhanced surface interaction with organic molecules. The tunable bandgap of CDs (and hence the specific photocatalytic degradation of different dyes) can be manipulated by altering the size, core, and functionalization. Light-harvesting capability due to band-gap-engineering-related applications result in CDs being used for degrading organic dyes under different wide range of light sources (i.e., sunlight, UV, visible, and NIR). The CDs combined with conventional photocatalyst procedures have demonstrated promising enhancement in the catalytic activity and reusability.

**Some possible limitations to overcome**: (1) Secondary contamination from CDs and their hybrid clusters' induced discharge into the environment. (2) Lack of efficiency of CDs in terms of its recovery and separation from aqueous solution may hinder their long-term reusability. (3) Interference with other contaminants, such as metals, pesticides, mud, and plastic particles, may minimize the interaction and access of active sites in the CDs (the case with most of the nanomaterials) to the organic dyes. (4) The pH-, time-, temperature-, and photo-induced-alterations-dependent studies are indispensable due to the possible variations in the optical properties of CDs. In addition, although most reported cases have generated reliable data to decompose only one dye at a time at the laboratory level, several organic dye combinations exist in water-contaminated places, and identifying individual dyes takes time and effort. It is essential to fabricate a photocatalyst that can degrade a combination of these contaminants simultaneously at the industrial level using natural resources (sunlight).

Moreover, realistic design of some experimental laboratory setup by replicating the naturally contaminated water system and evaluating different photocatalyst types (for specific and combination of organic dyes) is essential for the improvements in different materials that can be considered. Considering the chemical interactions between the photocatalyst, contaminant, and the final products of both photocatalyst and contaminant, along with the in-depth study to understand the underlying degradation mechanism, is essential. Well-organized studies across experimental and computational approaches are required to offer solid systematic evidence that will make the complex system understandable and boost the industry stakeholder’s confidence.

Nevertheless, based on the existing reports and ongoing extensive work from different groups on CDs-based photocatalysts, one can anticipate the resolution of all the above-mentioned disputes and some potential realistic scalable designs very soon.
